# Deployable machine learning-based decision support system for tracheostomy in acute burn patients

**DOI:** 10.1093/burnst/tkaf010

**Published:** 2025-05-13

**Authors:** Haisheng Li, Ni Zhen, Shixu Lin, Ning Li, Yumei Zhang, Wei Luo, Zhenzhen Zhang, Xingang Wang, Chunmao Han, Zhiqiang Yuan, Gaoxing Luo

**Affiliations:** Institute of Burn Research, Southwest Hospital, State Key Laboratory of Trauma and Chemical Poisoning, Third Military Medical University (Army Medical University), Chongqing 400038, China; Institute of Burn Research, Southwest Hospital, State Key Laboratory of Trauma and Chemical Poisoning, Third Military Medical University (Army Medical University), Chongqing 400038, China; School of Public Health, Zhejiang University School of Medicine, Hangzhou, Zhejiang 310009, China; Institute of Burn Research, Southwest Hospital, State Key Laboratory of Trauma and Chemical Poisoning, Third Military Medical University (Army Medical University), Chongqing 400038, China; Institute of Burn Research, Southwest Hospital, State Key Laboratory of Trauma and Chemical Poisoning, Third Military Medical University (Army Medical University), Chongqing 400038, China; Institute of Burn Research, Southwest Hospital, State Key Laboratory of Trauma and Chemical Poisoning, Third Military Medical University (Army Medical University), Chongqing 400038, China; The Second Affiliated Hospital of Zhejiang University School of Medicine, Hangzhou, Zhejiang 310009, China; The Second Affiliated Hospital of Zhejiang University School of Medicine, Hangzhou, Zhejiang 310009, China; The Second Affiliated Hospital of Zhejiang University School of Medicine, Hangzhou, Zhejiang 310009, China; Institute of Burn Research, Southwest Hospital, State Key Laboratory of Trauma and Chemical Poisoning, Third Military Medical University (Army Medical University), Chongqing 400038, China; Institute of Burn Research, Southwest Hospital, State Key Laboratory of Trauma and Chemical Poisoning, Third Military Medical University (Army Medical University), Chongqing 400038, China

**Keywords:** Burns, Tracheostomy, Decision support system, Artificial intelligence, Machine learning

## Abstract

**Background:**

Airway obstruction is a common emergency in acute burns with high mortality. Tracheostomy is the most effective method to keep patency of airway and start mechanical ventilation. However, the indication of tracheostomy is challenging and controversial. We aimed to develop and validate a deployable machine learning (ML)-based decision support system to predict the necessity of tracheostomy for acute burn patients.

**Methods:**

We enrolled 1011 burn patients from Southwest Hospital (2018–20) for model development and feature selection. The final model was validated on an independent internal cross-temporal cohort (2021, *n* = 274) and an external cross-institutional cohort (Second Affiliated Hospital of Zhejiang University School of Medicine 2020–21, *n* = 376). To improve the model’s deployment and interpretability, an ML-based nomogram, an online calculator, and an abbreviated scale were constructed and validated.

**Results:**

The optimal model was the eXtreme Gradient Boosting classifier (XGB), which achieved an AUROC of 0.973 and AUPRC of 0.879 in training dataset, and AUROCs of greater than 0.95 in both cross-temporal and cross-institutional validation. Moreover, it kept stable discriminatory ability in validation subgroups stratified by sex, age, burn area, and inhalation injury (AUROC ranging 0.903–0.990). The analysis of calibration curve, decision curve, and score distribution proved the feasibility and reliability of the ML-based nomogram, abbreviated scale (BETS), and online calculator.

**Conclusions:**

The developed system has strong predictive ability and generalizability in cross-temporal and cross-institutional evaluations. The nomogram, online calculator, and abbreviated scale based on ML show comparable prediction performance and can be deployed in broader application scenarios, especially in resource-limited clinical environments.

Highlights

- To our knowledge, this study is the first externally validated machine learning (ML)-based decision support system for tracheostomy, which includes an accurate ML model, and practical tools such as an AI-based nomogram, online calculator, and abbreviated scale (BETS score).
- The optimal ML model was the eXtreme Gradient Boosting classifier with six clinical features. The included features are basic clinical features, which can be directly obtained in clinical settings without expensive testing facilities.
- This decision support system could not only be quickly applied in bedside, but also be deployed in different levels of healthcare institutions and reduce regional disparities caused by medical resources.

## Background

Burns are the fourth most common type of accident worldwide and cause an estimated 180 000 deaths annually [1, 2]. Especially, the burden of burns is disproportionately shared globally, with about 90% of burns occurring in low- and middle-income countries (LMICs) [3]. The mortality rate of burns in LMICs is over 7 times higher than that of high-income countries [4]. For acute burn patients, the edema of skin tissue brought by large burns [5] and the airway destruction caused by inhalation injury can both lead to airway obstruction and even emergent asphyxia [6], which remains one of the leading causes of death for acute burn patients. Tracheostomy is the most effective and common method to keep patency of airway and start subsequent mechanical ventilation. About 30.9% of burn patients required tracheostomy [7] to ensure a safe airway, and that of burn patients with inhalation injury was up to 60.71% [6]. Delayed tracheostomy will increase the operational difficulties and the risk of serious complications, such as hemorrhage, and anoxemia. On the other hand, unnecessary tracheostomy will result in scar formation, pulmonary infection, and swallowing dysfunction. Therefore, timely and accurate tracheostomy is essential for burn patients.

However, the decision of tracheostomy for a burn patient requires a comprehensive and dynamic evaluation of clinical history and indications by a multidisciplinary expert team, which at least includes experienced specialists in burns, otolaryngology, and anesthesiology. Therefore, the decision is still empirical and subjective. It is difficult to determine the necessity of tracheostomy in time, especially for junior doctors with less clinical experience and institutions with limited medical resources. Although several guidelines for tracheostomy in the intensive care unit were developed in France [8], Danish [9], and Belgium [10], tracheostomy of burn patients is not within the scope of these guidelines. A Chinese guideline for tracheostomy in burn patients was published in 2018 [11], while the indications for tracheostomy were only generally covered, including deep burns in the head/face/neck, inhalation injury, and the possibility of tracheal stenosis or obstruction, most of which were obscure and could not be quantified. There is still a lack of an accurate and quantified strategy to help clinicians decide on tracheostomy for burn patients.

Artificial intelligence (AI) has been applied to develop various clinical decision-supporting systems, such as prediction of clinical efficacy [12] and cancer diagnosis [13, 14]. The AI-based decision support system can provide a real-time and numeric prediction of the clinical outcome, which will highly reduce subjectivity and provide standardized guidance in clinical practice. In the field of burns, AI models were mainly developed for the assessment of burn areas of different depths and mortality prediction [15, 16]. To our knowledge, there are only three AI-based tracheostomy predictions, and they were conducted for either ICU [17] or COVID-19 patients [18, 19]. However, none of them includes external validation and practical tools for direct clinical applications, and tracheostomy prediction in burn patients is less studied. In addition, the deployment of an AI-based decision support system in the clinical environment is challenging as the computation resources and clinicians’ AI experience are limited, especially in low-resource hospitals.

In this study, we developed a deployable machine learning (ML)-based decision support system for tracheostomy in burn patients and validated the system on large real-world clinical data from two large burn centers. It includes a ML model, an AI-based nomogram, an online calculator, and an abbreviated scale. This system could provide accurate and quantified guidance for clinicians on the decision of tracheostomy in burn patients and can be easily deployed in different clinical environments.

## Methods

### Study design and populations

This retrospective cohort study was conducted at the Southwest Hospital of the Third Military Medical University (Hospital A), and the Second Affiliated Hospital of Zhejiang University School of Medicine (Hospital B) with institutional review board approvals (No. KY2021121 and No. IR2022185, respectively). Written informed consent from the participants was not required to participate in this study following the national legislation and the institutional requirements.

**Figure 1:**
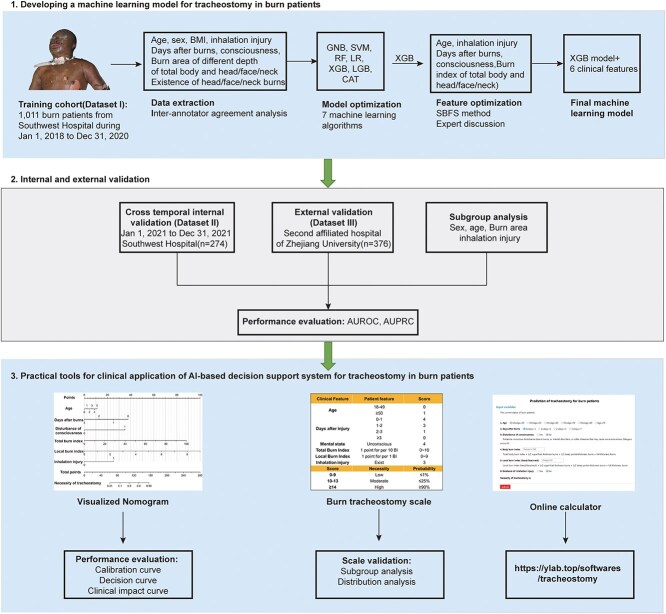
Workflow of ML based decision support system for tracheostomy in burn patients

We enrolled burn patients with the following inclusion criteria: (i) age 18 years or older, (ii) admitted earlier than 7 days after burned, (iii) injured by scald, flame burns, electrical burns, and other acute burns; Exclusion criteria: (i) patients already underwent tracheostomy or endotracheal intubation when admission, (ii) patients rejected tracheostomy, (iii) patients without complete data required for model development. The decision support system was developed based on eligible patients from Hospital A between 1 January 2018 and 31 December 2020 (Dataset I). Eligible patients from Hospital A (1 January 2021–31 December 2021, Dataset II) and Hospital B (1 January 2020–31 December 2021, Dataset III) were collected for cross-temporal and cross-institutional validation, respectively. Figure 1 visualizes the workflow of this study.

### Data collection and processing

The following data were extracted from electronic health records of the Burn Specific Database of Hospital A and B, including (i) demographic features: age, sex, BMI; (ii) admission status: days after burns, disturbance of consciousness; (iii) burn severity: burn area of different depths of total body and head/face/neck (superficial partial-thickness burns, deep partial-thickness burns, and full-thickness burns), existence of head burns, existence of facial burns, existence of neck burns, existence of inhalation injury. To be simplified, all the cases without normal consciousness, such as coma, mania, dementia and other mental disorders, were defined as disturbance of consciousness.

The outcome is whether the patient has been performed a tracheostomy or not during hospitalization. Briefly, the clinical decision of tracheostomy should be considered once a patient had one of the following cases: burns on face/neck/head, edema on face/neck/head, possibility or existence of airway obstruction, severe burns in early-stage, inhalation injury, ARDS or respiratory failure, disturbance of consciousness, according to the expert consensus on tracheostomy and intubation for burn patients [11]. Then, the decision of tracheostomy was determined by a multidisciplinary expert team, including senior doctors of burns, otolaryngology, and anesthesiology. Percutaneous tracheostomy was the first-line method, followed by open surgical tracheostomy. After the enrolled population was determined, the necessity of tracheostomy was confirmed again by a discussion of experienced experts.

During data collection, all the included patients were randomly and blindly assigned to two independent investigators (Yumei Zhang and Wei Luo for Hospital A, and Zhenzhen Zhang and Shixu Lin for Hospital B) and were examined by another researcher when conflicts occurred (Ning Li for Hospital A, and Xinggang Wang for Hospital B). To evaluate the inter-annotator agreement, 100 patients were randomly selected and annotated by two investigators independently, and the overall agreement of all indicators between them achieved 97.64%.

### Model development

We implemented seven state-of-the-art ML algorithms, which have been widely applied in the development of clinical decision-making support systems and demonstrated exceptional performance [20–22]. Specifically, these include the Naive Gaussian Bayes classification model (GNB), logistic regression (LR), support vector machine (SVM) [23], random forest (RF) [24], eXtreme Gradient Boosting classifier (XGB) [25], Light Gradient Boosting Machine (LGB) [26], CatBoost (CAT) [27]. Those algorithms were evaluated through the area under the receiver operating characteristic curve (AUROC) and the area under the precision-recall curve (AUPRC). While ensemble learning can enhance performance by combining multiple models, we do not incorporate it into our study due to deployment and tool development considerations. Continuous features such as age, BMI, and days after burns were scored according to the criteria in Supplementary Table 1.

To make full use of the limited clinical data, cross-validation was adopted to train and evaluate the model performance in Dataset I. Meanwhile, grid-search was utilized to optimize the hyper-parameters of algorithms. In particular, we employed model-specific strategies to mitigate dataset imbalance. For instance, we adjusted the weight of the minority class during training by setting relevant parameters (e.g. class weight in LR), enabling the models to appropriately address minority classes and prevent inherent bias. The sequential backward floating selection (SBFS) [28] was conducted for feature selection, which independently trains and evaluates a model with a subset of features and gradually removes the least valuable feature to search for a better feature set. We chose AUPRC as the evaluation index of SBFS to search for the optimal feature subset. Considering a real-world clinical scenario, a patient who should but does not receive a timely tracheostomy may suffer from a more serious outcome, i.e. a false negative is far costlier than a false positive from the perspective of the model prediction. Therefore, we took the F_β_score = [(1 + *β^2^*)*(precision*recall)]/(β^2^*precision+ recall) and set the *β* as 2 to search the hyper-parameters combination with a higher recall. The model with the best performance on both AUROC and AUPRC was selected as the optimum algorithm. The result of feature selection was confirmed by an expert team to determine the final feature subset S_final_. Together, the final ML model was constructed based on the optimum algorithm and feature subset S_final_.

### Model validation and subgroup analysis

The final model performance was validated in the internal cross-temporal dataset II and external cross-institutional dataset III, respectively. Moreover, the model was evaluated under subgroup cohorts to test its stability and sensitivity.

Subgroups were stratified by several clinical factors commonly used in burn research, including sex, age, TBSA (Total Body Surface Area (%)) burns, and inhalation injury. Considering the limited sample size of subgroups, Dataset II and Dataset III were merged in this validation. AUROC was calculated to assess the final model performance.

### Development of clinical nomogram

To improve the model’s availability and interpretability in clinical practice, we converted our model into a visualized nomogram. The coefficient of each factor in the ML model was directly used to construct the variable axes in the nomogram, which visually presents each factor’s contribution to the final prediction. We used the rms *package* in R software to build the nomogram model.

### Development of the abbreviated burn early tracheostomy scale (BETS) and the online calculator

To simplify the application complexity in clinical practice, the BETS was developed based on the nomogram and an online calculator for fast calculating patient risk. The score range of each feature is calculated approximately by one-tenth of its point in the developed nomogram. We set the threshold to split the burn patients into low-risk, moderate-risk, and high-risk subgroups according to tracheostomy risk in different score segments. Then, we calculated the proportion and distribution of tracheostomy and non-tracheostomy of different subgroups in the training dataset (Dataset I) and validation dataset (Dataset II and III) discriminated by the BETS.

### Statistical analysis

Descriptive statistics were reported as frequencies and proportions for categorical variables, and mean and SD for continuous variables. The Chi-square test was applied to assess significant associations between categorical variables (frequency and percentage) and the t-test was used to compare two means of continuous variables. *P* values < .05 were considered statistically significant.

To compare different ML algorithms, the AUROC and AUPRC of three-fold cross-validations on Dataset I were calculated. This process was repeated 10 times, and the mean AUROC and AUPRC values with SD were reported. The clinical feasibility of the nomogram was evaluated quantitatively by calibration curve [29], decision curve [30–32], net benefit [32], and clinical impact curve [31]. The net benefit is defined as the proportion of true positives minus the proportion of false positives and standardized by the relative harm of a false-positive and false-negative result. Furthermore, a clinical impact curve was created to illustrate the estimated number of high-risk patients at different risk thresholds and visually shows the proportion of those who are tracheostomy cases (true positive). Bootstraps of 1000 resample (with replacement) were set and corresponding metrics with 95% CIs were calculated in different validation datasets, respectively.

The complete pipeline that encompasses data processing, feature selection, model training and optimization, internal and external validation, and the creation of practical tools (i.e. nomogram, brief scale, and online calculator), is developed on Python version 3.9.9 and R version 4.2.0. All code is publicly available on GitHub (https://github.com/LiHaisheng-burns/tracheostomy-burn). We utilized standard libraries to ensure the stability and reproducibility of our models, including scikit-learn [33], XGBoost [25], LightGBM [26], and CAT [27], as well as rms [34] and rmda [35] for developing nomograms, calibration curves, and decision curves.

## Results

### Patient characteristics

We initially enrolled 5313 burn patients from Southwest Hospital from January 2018 to December 2021, and 1400 burn patients from the Second Affiliated Hospital of Zhejiang University School of Medicine between January 2020 and December 2021. After step-by-step selection, 1285 eligible patients from Southwest Hospital and 376 from the Second Affiliated Hospital of Zhejiang University School of Medicine were finally included (Supplementary Figure 1**)**. Inpatient demographics and hospital characteristics are listed in Table 1. The training, cross-temporal validation, and external cross-institutional validation datasets had similar characteristics. Dataset I, Dataset II, and Dataset III included 1011 patients, 274 patients, and 376 patients, with the percentage of tracheostomy of 11.47%, 12.41%, and 14.36%, respectively (*P* = .3453). Compared to patients without tracheostomy, patients with tracheostomy had an earlier admission day after burns, a higher percentage of inhalation injury, burns on the head/face/neck, and more TBSA and head/neck/face.

**Table 1:** Population characteristics

Categories	Hospital A								Hospital B				*P* value^*^
	Training Dataset I (2018–20)				Internal validation Dataset II (2021)				External validation Dataset III (2020–21)				
	Total (*n* = 1011)	With Tracheotomy (*n* = 116)	Without Tracheotomy (*n* = 895)	*P* value	Total (*n* = 274)	With Tracheotomy (*n* = 34)	Without Tracheotomy (*n* = 240)	*P* value	Total (*n* = 376)	With Tracheotomy (*n* = 54)	Without Tracheotomy (*n* = 322)	*P* value	
Sex													0.798
Male	768(75.96%)	92(79.31%)	676(75.53%)	0.4196	203(74.09%)	29(85.29%)	174(72.5%)	0.5856	290(76.06%)	44(81.48%)	242(75.16%)	0.3897	
Female	243(24.04%)	24(20.69%)	219(24.47%)		71(25.91%)	5(14.71%)	66(27.5%)		90(18.52%)	10(24.84%)	80(23.94%)		
Age, years (Mean ± SD)	44.86 ± 14.67	48.43 ± 15.11	44.39 ± 14.56	0.0053	46.43 ± 13.90	49.00 ± 15.34	46.10 ± 13.99	0.2648	47.20 ± 16.10	47.98 ± 17.14	47.07 ± 15.95	0.7003	0.019
BMI (Mean ± SD)	23.77 ± 3.70	24.10 ± 3.56	23.72 ± 3.72	0.2999	24.01 ± 3.35	24.37 ± 3.98	23.85 ± 3.46	0.4271	23.41 ± 3.26	23.96 ± 3.39	23.31 ± 3.24	0.1778	0.301
Admission days after burns (Mean ± SD)	1.13 ± 1.64	0.22 ± 0.54	1.23 ± 1.73	<0.0001	0.76 ± 1.07	0.21 ± 0.54	1.38 ± 1.71	<0.0001	0.76 ± 1.30	0.33 ± 0.58	0.83 ± 1.37	0.0098	<0.01
Burn etiology				<0.001				<0.001				<0.001	<0.001
Flame Burns,	462(45.70%)	95(81.90%)	367(41.01%)		114(41.61%)	21(61.76%)	93(38.75%)		170(45.21%)	28(51.85%)	142(44.10%)		
Electric burns	227(22.45%)	2(1.72%)	225(25.14%)		74(27.01%)	8(23.53%)	66(27.5%)		47(12.50%)	2(3.70%)	45(13.98%)		
Explosion	30(2.97%)	3(2.59%)	27(3.02%)		8(2.92%)	4(11.76%)	4(1.67%)		31(8.24%)	22(40.74%)	9(2.80%)		
Chemical burns	48(4.75%)	0(0)	48(5.36%)		9(3.28%)	0(0)	9(3.75%)		17(4.52%)	1(1.85%)	16(4.97%)		
Scalds	175(17.31%)	10(8.62%)	165(18.44%)		51(18.61%)	0(0)	51(21.25%)		77(20.48%)	1(1.85%)	76(23.60%)		
Others	69(6.82%)	6(5.17%)	63(7.04%)		18(2.94%)	1(7.08%)	17(6.57%)		34(9.04%)	0(0)	34(10.56%)		
Inhalation injury	137(13.55%)	84(72.41%)	53(5.92%)	<0.0001	72(26.28%)	30(88.24%)	42(17.5%)	<0.0001	57(15.16%)	40(74.07%)	17(5.28%)	<0.0001	<0.001
Total burn area (TBSA%, Mean ± SD)	18.14 ± 18.79	48.54 ± 28.69	14.19 ± 12.56	<0.0001	27.66 ± 23.52	51.85 ± 27.91	15.82 ± 13.64	<0.0001	21.57 ± 23.19	53.69 ± 30.44	16.18 ± 16.52	<0.0001	0.183
Total Full thickness burn area (Mean ± SD)	5.26 ± 11.93	23.57 ± 23.47	2.89 ± 6.40	<0.0001	8.38 ± 15.16	24.00 ± 20.53	3.24 ± 6.31	<0.0001	6.98 ± 10.62	27.94 ± 30.91	3.47 ± 7.30	<0.0001	0.152
Total burn index (Mean ± SD)	11.70 ± 14.45	36.06 ± 24.73	8.54 ± 8.38	<0.0001	18.02 ± 18.57	37.93 ± 23.06	9.49 ± 8.83	<0.0001	14.28 ± 18.26	40.81 ± 29.15	9.83 ± 10.53	<0.0001	0.188
Head/neck/face burns	622(61.52%)	111(95.69%)	511(57.09%)	<0.0001	180(65.69%)	32(94.12%)	148(61.67%)	<0.0001	225(59.84%)	52(96.30%)	173(53.73%)	<0.0001	0.301
Local burn area (TBSA%, Mean ± SD)	2.55 ± 2.55	6.00 ± 2.37	2.10 ± 2.20	<0.0001	5.15 ± 1.82	6.44 ± 2.39	2.31 ± 2.26	<0.0001	2.57 ± 2.75	6.16 ± 2.32	1.97 ± 2.32	<0.0001	0.254
Local burn index (Mean ± SD)	1.38 ± 1.52	3.71 ± 1.99	1.08 ± 1.15	<0.0001	2.75 ± 1.28	3.71 ± 1.99	1.08 ± 1.15	<0.0001	1.37 ± 1.59	3.60 ± 1.88	1.00 ± 1.18	<0.0001	0.293
Specific burn sites													
Head	264(26.11%)	85(73.28%)	179(20%)	<0.0001	74(27.01%)	26(76.47%)	48(20%)	<0.0001	68(18.09%)	27(50%)	41(12.73%)	<0.0001	0.005
Neck	499(49.36%)	96(82.76%)	403(45.03%)	<0.0001	148(54.01%)	31(91.18%)	117(48.75%)	<0.0001	163(43.35%)	48(88.89%)	115(35.71%)	<0.0001	0.023
Face	581(57.47%)	110(94.83%)	471(52.63%)	<0.0001	171(62.41%)	31(91.18%)	140(58.33%)	0.0001	217(57.71%)	52(96.30%)	165(51.24%)	<0.0001	0.348
Disturbance of Consciousness	22(2.18%)	17(14.66%)	5(0.56%)	<0.0001	3(1.09%)	3(8.82%)	0(0)	0.0018	9(2.39%)	6(11.11%)	3(0.93%)	0.0004	0.461

Hospital A: Southwest Hospital of the Third Military Medical University. Hospital B: Second Affiliated Hospital of Zhejiang University School of Medicine. *BMI* body mass index, *TBSA* total body surface area. ^*^comparison among three datasets

### Model development

**Figure 2:**
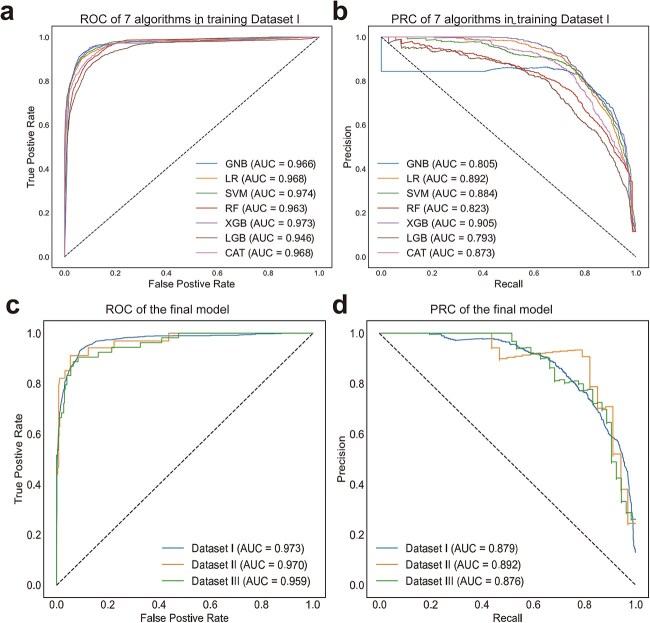
Performance of seven ML algorithms and the final model

The performance of all ML algorithms with 15 clinical features in training Dataset I was compared to select the best ML algorithm. As shown in Figure 2a and 2b and Table 2, all algorithms basically achieved good performance. The XGB algorithm reached the second-highest AUROC of 0.973 (SD 0.121) and the highest AUPRC of 0.905 (SD 0.022). Therefore, the XGB algorithm was selected as the optimum algorithm to conduct subsequent analysis.

**Table 2:** Performance of different ML algorithms in training dataset I

Algorithm	AUROC		AUPRC	
	Mean	SD	Mean	SD
GNB	0.966	0.013	0.805	0.057
LR	0.968	0.012	0.892	0.027
SVM	0.974	0.008	0.884	0.024
RF	0.963	0.009	0.823	0.033
XGB	0.973	0.012	0.905	0.022
LGB	0.946	0.016	0.793	0.040
CAT	0.968	0.011	0.873	0.028

*AUROC* Areas Under the Receiver Operator Characteristic curve, *AUPRC* Areas Under the Precision-Recall Curve, *GNB* Naive Gaussian Bayes classification model, *LR* logistic regression, *SVM* support vector machine, *RF* random forest. *XGB* eXtreme Gradient Boosting classifier, *LGB* Light Gradient Boosting Machine, *CAT* CatBoost

Experiments on feature selection show that the feature subset with 10 features achieved the highest AUPRC (0.914 [SD 0.020]) (Supplementary Figure 2), where the corresponding AUROC is 0.977 (SD 0.011). The selected features include age, days after burns, disturbance of consciousness, TBSA of different depths (superficial partial-thickness burns, deep partial-thickness burns, full-thickness burns), local burn area in different depths (superficial partial-thickness burns, local deep partial-thickness burns, local full-thickness burns), and the existence of inhalation injury (Supplementary Table 2). Following the clinical practice [36, 37], we introduced the burn index (1/2 superficial partial-thickness burns +1/2 deep partial-thickness burns + full-thickness burns), a widely used index for estimating burns severity in burn care [38], to integrate the burned area of different depths. Both the total (TBSA) burn index and local burn index (head/face/neck region) were used to merge the separated features of the burned area of different depths, resulting in the final feature set S_final_ with six features (age, days after burns, disturbance of consciousness, total burn index, local burn index, and inhalation injury). The XGB model with S_final_ was retrained in Dataset I to develop the final model, which reached an AUROC of 0.973 (SD 0.012) and AUPRC of 0.879 (SD 0.030).

### Model validation

The final XGB model was externally validated in Dataset II and Dataset III. Results are shown in Figure 2c and 2d. It achieved a good performance in both validation datasets, with the AUROC of 0.970 (95%CI 0.969–0.971) and AUPRC of 0.892 (0.890–0.895) in Dataset II, and the AUROC of 0.959 (0.958–0.960) and AUPRC of 0.876 (0.874–0.878) in Dataset III.

### Subgroup analysis

Further subgroup analysis was conducted to test the stability and sensitivity of the final model. As shown in Table 3 and Supplementary Figure 3, the AUROCs of different subgroups ranged from 0.903 to 0.990. The strong discrimination ability of the model was maintained when the cohort was stratified by sex, age, total burn area, and inhalation injury.

**Table 3:** Subgroup performance on validation datasets

Subgroup	Sample size (*n*)	AUROC	
		Mean	95%CI
Sex			
Male	161	0.968	0.967–0.969
Female	489	0.962	0.962–0.963
Age (years)			
18–29	93	0.935	0.933–0.937
30–39	132	0.983	0.982–0.983
40–49	130	0.990	0.990–0.991
50–59	185	0.977	0.976–0.977
60–69	59	0.952	0.949–0.954
≥70	51	0.903	0.899–0.907
Total burn area (%TBSA)			
0–30	480	0.936	0.935–0.938
30–50	101	0.948	0.947–0.950
50–100	69	0.930	0.928–0.932
Inhalation injury			
Yes	129	0.912	0.910–0.913
No	521	0.904	0.901–0.906

*AUROC* area under the receiver operating characteristic curve, *TBSA* total body surface area

### The development and performance of XGB-based nomogram

**Figure 3:**
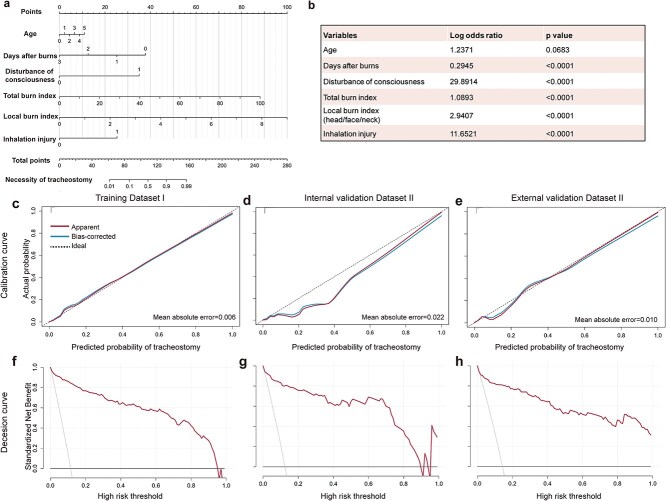
Development and validation of ML -based nomogram for predicting the necessity of tracheostomy in burn patients

A nomogram was created to visualize the final XGB model (Figure 3a) and directly illustrate the impact of every clinical feature. Clinicians can calculate the total score of six features according to the patient status and then determine the necessity of tracheostomy by mapping the score in the last caliper. The odds ratio of each predictor and *P*-value were also calculated (Figure 3b). The risk factors for tracheostomy prediction were older age (*P* = .0683), shorter days after burns (*P* < .0001), disturbance of consciousness (*P* < .0001), higher total body burn index (*P* < .0001), higher local burn index (*P* < .0001) and existence of inhalation injury (*P* < .0001).

The clinical benefit of the nomogram was evaluated with the calibration curve and decision curve. As shown in Figure 3c–e, the calibration curves roughly overlapped with the ideal lines in the training and validation datasets, showing strong agreement of the predicted probabilities with actual observations (mean absolute errors: 0.006, 0.022, and 0.010 in Dataset I, Dataset II, and Dataset III, respectively). These decision curves showed a high net benefit of applying the developed nomogram in clinical practice (Figure 3f–h). Threshold probabilities for the standardized net benefit associated with the application of the nomogram in deciding tracheostomy ranged from 0.00 to 0.95 in Dataset I, 0.00 to 0.89 in Dataset II, and 0.00 to 1.00 in Dataset III. The corresponding clinical impact curve presented a high proportion of true cases in predicted high-risk patients, implying a high clinical practical value of our nomogram **(**Supplementary Figure 4**)**.

### The development and performance of BETS

**Figure 4:**
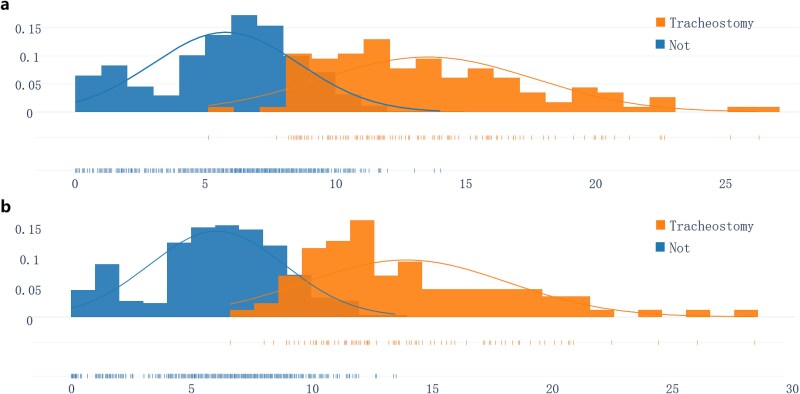
The score distribution based on BETS score

The detailed setting of BETS is shown in Table 4. Burn patients can be stratified into three groups with different tracheostomy probabilities based on BETS score: low-risk (score: 0–7, tracheostomy probability ≤1%), moderate risk (score: 8–12, tracheostomy probability ≤30%), and high-risk (score: ≥13, tracheostomy probability ≥90%). The tracheostomy risk showed a similar distribution between predicted and actual groups (Table 5). The BETS score distribution in the training and validation dataset (Figure 4) showed a strong discriminant capability between tracheostomy and non-tracheostomy patients and a high consistency among different datasets, demonstrating the feasibility and reliability of BETS. The overview of the online calculator (https://lihaisheng-burns.github.io/tracheostomy-burn/) is shown in Supplementary Figure 5.

**Table 4:** The abbreviated burn early tracheostomy scale (BETS)

Clinical feature	Patient status	Score
Age	18–49	0
	≥50	1
Days after burns	0–1	4
	1–2	3
	2–3	1
	≥3	0
Consciousness	Unconscious	4
Total Burn Index	1 point per 10 BI	0 ~ 10
Local Burn Index	1 point per 1 BI	0 ~ 9
Inhalation injury	Exist	3
Total score	Necessity	Probability
0–7	Low	≤1%
8–12	Moderate	≤30%
≥13	High	≥90%

Total body burn index, 1/2 superficial thickness burns on total body +1/2 deep partial-thickness burns on total body + full-thickness burns on the total body. Local burn index, 1/2 superficial thickness burns on head, face, and neck +1/2 deep partial-thickness burns on head, face, and neck + full-thickness burns on head, face, and neck. The mental or conscious disturbance means the patient had conscious disturbance due to burns or neurological diseases or had mental diseases or other diseases that may cause unconsciousness (Glasgow score < 9)

**Table 5:** Probability of tracheostomy in subgroups with different scores in training dataset (dataset I) and validation dataset (dataset II/III)

Score	Necessity	Estimated probability	Actual probability (Training Dataset)	Actual probability (Validation Datasets)
0–7	Low	≤1%	0.69%	0.23%
8–12	Moderate	≤30%	25.99%	27.98%
≥13	High	≥90%	94.55%	95.24%

## Discussion

The indications and timing of tracheostomy are highly disputed [39], and the tracheostomy decisions are mainly determined by a multidisciplinary and experienced expert team after thorough discussions. This empirical-based clinical decision is subjective and difficult. Although a few relevant studies aimed at tracheostomy evaluation, they still lacked external validation in model development and practical tools for clinical application [7, 17, 18, 40–42]. To our knowledge, this study is the first externally validated ML-based decision support system for tracheostomy, simultaneously including a ML model, a nomogram, an online calculator, and an abbreviated scale, which could aid the timely tracheostomy decision-making for burn patients.

Traditional risk factor determination and scoring systems rely on clinical experience or statistical methods [38], which have limited predicting ability. In this study, we investigated multiple cutting-edge ML algorithms to develop a robust clinical scoring system. Although several algorithms achieved fine performance, performance variations across them were observed due to the difference in dataset adaptability and parameter tuning. From model development to practical tools, the proposed methodology in our study is mainly driven by clinical data. It could automatically and efficiently integrate many clinical features without predefined statistical assumptions and stepwise statistical analysis. Through model hyper-parameter optimization and performance comparison, this study adopted XGB model with the linear booster and then directly constructed a visualized nomogram with its model coefficients rather than re-training a logistic regression model. Compared with a tree-based classifier, which is commonly used in XGB, the linear booster improves model interpretability and lays a foundation for nomogram construction. Compared with traditional logistic regression, our XGB model adopted boosting, an ensemble learning strategy, to train many weak linear classifiers with more strict regularization to avoid overfitting and then integrate them to develop a robust ensemble model.

The developed XGB model and nomogram showed a strong discriminant ability, stability, and feasibility. First, the AUROCs and AUPRCs of the XGB model in training and validation datasets were higher than 0.90, which outperforms previous studies of tracheostomy prediction for patients of ICU [17] and COVID-19 [18]. Second, we stratified the validation datasets into different subgroups by risk factors commonly concerned in burn field, including sex, age, TBSA burned, and inhalation injury. The strong discriminant ability of the model was stably maintained in these cohorts. Third, the calibration curves of the nomogram showed the consistency between predictive probability and actual risk, and the decision curves have a broad range of thresholds for high net benefit, both demonstrating the feasibility and positive clinical impact of our model. However, this model was developed based on the retrospective data and need further prospective validation and modification in future.

A total of six clinical features were included in the final model, ranked by their importance: local burn index in head/neck/face, total body burn index, days after burns, disturbance of consciousness, inhalation injury, and age. Among them, total body burn index, inhalation injury, and age have been recognized as potential clinical indications for tracheostomy in burns [11, 43]. The inhalation of smoke, heat, toxicants, and chemicals could directly destroy the airway and gradually lead to swelling, increased sputum, and falling of necrotizing epithelium, which could easily lead to airway obstruction [44]. Thus, inhalation injury contributes to the main factors for considering tracheostomy. The burn index, assessed by area and depth, can directly represent the burn severity. Patients with a high burn index typically exhibit high stress, inflammation, and edema in almost all organs [45]. Additionally, older age is recognized as a risk factor for mortality in burns [46]. The skin tissue of elders is relatively loose, and their compensatory capacity for organ functions is weak, making them more susceptible to requiring mechanical ventilation than younger individuals [47]. Furthermore, days after burns, local burn index, and disturbance of consciousness were less defined and investigated in previous studies [7, 48]. The days after burns presents the dynamic progress of burns. The main pathophysiologic changes brought by burns, including increased permeability of vascular, subsequent fluid leakage, and induced edema, occur immediately after burns, peak at 6–8 hours, and last 48–72 hours [49]. Therefore, the risk of airway obstruction caused by edema increases rapidly within the day of burns and then decreases gradually with time. The local burn index reflects the severity of local burn and denotes the local swelling in the head/face/neck region, which is likely to result in laryngeal edema and emergent asphyxia [6]. Unconsciousness, especially the delirium caused by severe burns [50] and the shock caused by toxication or serious infection, may result in loss of breathing capacity, and airway obstruction due to pulmonary aspiration of gastric contents. Hence more aggressive treatments will be needed in this situation [51]. Meanwhile, five clinical features, including sex, BMI, the existence of head burns, the existence of facial burns, and the existence of neck burns, were not included in the final model due to their limited impact on improving model performance after SBFS. This may be attributed to the fact that the integrated local burn index more accurately and comprehensively reflects the severity of burns in the head/face/neck regions compared to simply noting the presence of burns in these areas. As a whole, the clinical features identified by our decision support system closely fit the characteristics of burn clinical scenarios.

The empirical-based clinical decision-making is limited by the capabilities and experience of clinicians and hence has poor generalizability, as the training of an experienced clinician requires large numbers of clinical resources and relies heavily on the clinical environment and platforms. In contrast, our ML-based model develops based on objective indicators and provides accurate and quantified guidance for all clinicians. Moreover, the three auxiliary tools—nomogram, abbreviated scale, and online calculator, can further improve the accessibility of our system. This is particularly beneficial in many hospitals, especially those with limited medical resources, where deploying an AI-based model is challenging due to scarce computing resources and the need for AI training. The fast deployment of this data-driven decision support system can reduce the disparities across different clinical institutions, as the gap between resource-rich and resource-poor institutes can be narrowed through our AI tools.

Given the high accessibility of included clinical features, this decision-support system is expected to benefit primary clinicians in burn units and regions with limited medical resources. Meanwhile, the final decision on tracheostomy should consider the AI predictions in combination with additional clinical information, including symptoms, oxygenation status, airway detection, and the patient’s opinion. Additionally, more clinical examinations should be integrated with this system to enhance the efficiency and precision of tracheostomy decisions. For instance, while bronchoscopy is the gold standard for diagnosing inhalation injury and airway injury, it requires multidisciplinary collaboration and is usually inapplicable in emergent and resource-constrained settings. Sometimes, the early changes observed via bronchoscopy are not sufficiently pronounced to meet the criteria of tracheostomy. Considering the potentially fatal consequences of failure to tracheostomy after edema or injury deterioration, a preventive tracheostomy is usually recommended in clinical practice. [52]. Therefore, the ML-based nomogram, online calculator, or brief scale can be employed to identify acute burn populations at high risk of tracheostomy under all circumstances. The developed decision support system is open-loop. Subsequently, if feasible, bronchoscopy and other clinical examinations should be performed to confirm the necessity of tracheostomy. Recently, bronchoscopy has also been used to guide percutaneous dilational tracheostomy to ensure more accurate placement of the tube and reduce complications [53]. Furthermore, dynamic evaluation of the necessity for tracheostomy is important, especially for patients with a moderate risk of requiring tracheostomy. However, it is difficult to directly quantify the extent of swelling in the airway and surrounding tissues. Based on the kinetics of swelling during the early stage of burns [49], we chose the days after burns as the preliminary dynamic indicator for burn progression and included it in the final model. Therefore, to ensure accurate and timely decisions regarding tracheostomy, future studies are necessary to incorporate more dynamic features, such as SpO2, local skin tension, and blood flow [11].

There are several potential limitations in our study. First, the model developed in this study is based on the burn cohorts primarily consisting of Chinese patients and requires further validation in populations of different races. However, the overall methodology is easily replicable and evaluated on different cohorts, as our code and models are publicly available. Second, as this work is designed to serve broad clinical scenarios, some clinical examinations (e.g. laryngoscope or bronchoscope) that need advanced medical instruments were not included in our model, which may limit model performance. Nonetheless, our system tried to use the most accessible features to ensure generalizability. Third, patient mortality does not correspond exclusively to tracheostomy, due to the systemic burden and long treatment period caused by severe burns. Therefore, a prospective clinical trial and extension to dynamic evaluation are needed to examine and improve the clinical benefit of the proposed approach.

## Conclusions

We constructed the first externally validated ML-based decision support system for tracheostomy in burn patients. Through cross-temporal and cross-institutional validation, the developed system demonstrated high performance and strong generalizability. Moreover, multiple practical tools in this system, including the nomogram, online calculator, and abbreviated scale, showed good clinical feasibility. This system can accurately and efficiently estimate the tracheostomy necessity and is promising to alleviate the dilemma of difficult decision-making of tracheostomy, especially for the regions with limited medical resources. The development of this system provided a practical paradigm for the development of an AI-based decision support system for clinical scenarios.

## Supplementary Material

Supplementary_Materials_tkaf010(1)

## Data Availability

The datasets used and/or analyzed during the current study are available from the corresponding and first author upon reasonable request.
