# Supplementary material for: Deployable machine learning-based decision support system for tracheostomy in acute burn patients
Source: Burns Trauma. 2025 May 13;13:tkaf010. doi: 10.1093/burnst/tkaf010 (PMC12070481; doi:10.1093/burnst/tkaf010)
Supplement: Supplementary_Materials_tkaf010(1) [file supplementary_materials_tkaf010(1).docx]

# Supplementary Materials

**1. Supplementary Tables**

**1.1 Supplementary Table 1:** Segmentation criteria of continuous features

**1.2 Supplementary Table 2:** AUPRC of XGB algorithm with different feature subsets by SBFS

**2. Supplementary Figures**

**2.1 Supplementary Figure 1:** The flowchart of patient inclusion and exclusion

**2.2 Supplementary Figure 2:** AUPRC of XGB algorithm with different feature subsets by SBFS

**2.3 Supplementary Figure 3:** Comparative performances of the final model in different subgroups stratified by sex, age, total burn area, and inhalation injury

**2.4 Supplementary Figure 4:** The clinical impact curve of the developed nomogram

**2.5 Supplementary Figure 5:** The developed online calculator

## 1. Supplementary Tables

**Supplementary Table 1. Segmentation criteria of continuous features**

| Age | | BMI | | Days after burns | |
| --- | --- | --- | --- | --- | --- |
| 18≤x≤29 | 0 | x≤24 | 0 | 0≤x<1 | 0 |
| 30≤x≤39 | 1 | 24≤x<28 | 1 | 1≤x<2 | 1 |
| 40≤x≤19 | 2 | x≥28 | 2 | 2≤x<3 | 2 |
| 50≤x≤59 | 3 |  |  | 3≤x<7 | 3 |
| 60≤x≤69 | 4 |  |  |  |  |
| x≥70 | 5 |  |  |  |  |

**Supplementary Table 2. AUPRC of XGB algorithm with different feature subsets by SBFS**

| **AUPRC-Mean** | **AUPRC-SD** | **Feature subsets** |
| --- | --- | --- |
| 0.9142 | 0.0201 | 'Disturbance of consciousness', 'superficial partial thickness burns', 'deep partial thickness burns', 'full thickness burns', 'local_superficial partial thickness burns', 'local_deep partial thickness burns', 'local_full thickness burns', 'inhalation injury', 'age', 'days after burns' |
| 0.9134 | 0.0204 | 'Disturbance of consciousness', 'deep partial thickness burns', 'full thickness burns', 'local_deep partial thickness burns', 'local_full thickness burns', 'inhalation injury', 'age', 'days after burns' |
| 0.9127 | 0.0201 | 'Disturbance of consciousness', 'superficial partial thickness burns', 'deep partial thickness burns', 'full thickness burns', 'head', 'local_superficial partial thickness burns', 'local_deep partial thickness burns', 'local_full thickness burns', 'inhalation injury', 'age', 'days after burns' |
| 0.9122 | 0.0196 | 'Disturbance of consciousness', 'superficial partial thickness burns', 'deep partial thickness burns', 'local_superficial partial thickness burns', 'local_deep partial thickness burns', 'local_full thickness burns', 'inhalation injury', 'age', 'days after burns' |
| 0.9109 | 0.0233 | 'Disturbance of consciousness', 'deep partial thickness burns', 'full thickness burns', 'local_deep partial thickness burns', 'local_full thickness burns', 'inhalation injury', 'days after burns' |
| 0.9103 | 0.0200 | 'Disturbance of consciousness', 'superficial partial thickness burns', 'deep partial thickness burns', 'full thickness burns', 'head', 'neck', 'local_superficial partial thickness burns', 'local_deep partial thickness burns', 'local_full thickness burns', 'inhalation injury', 'age', 'days after burns' |
| 0.9090 | 0.0240 | 'Disturbance of consciousness', 'deep partial thickness burns', 'local_deep partial thickness burns', 'local_full thickness burns', 'inhalation injury', 'days after burns' |
| 0.9085 | 0.0201 | 'Disturbance of consciousness', 'superficial partial thickness burns', 'deep partial thickness burns', 'full thickness burns', 'head', 'face', 'neck', 'local_superficial partial thickness burns', 'local_deep partial thickness burns', 'local_full thickness burns', 'inhalation injury', 'age', 'days after burns' |
| 0.9066 | 0.0211 | 'Disturbance of consciousness', 'superficial partial thickness burns', 'deep partial thickness burns', 'full thickness burns', 'head', 'face', 'neck', 'local_superficial partial thickness burns', 'local_deep partial thickness burns', 'local_full thickness burns', 'inhalation injury', 'age', 'BMI', 'days after burns' |
| 0.9041 | 0.0223 | 'sex', 'Disturbance of consciousness', 'superficial partial thickness burns', 'deep partial thickness burns', 'full thickness burns', 'head', 'face', 'neck', 'local_superficial partial thickness burns', 'local_deep partial thickness burns', 'local_full thickness burns', 'inhalation injury', 'age', 'BMI', 'days after burns' |
| 0.8920 | 0.0274 | 'deep partial thickness burns', 'local_deep partial thickness burns', 'local_full thickness burns', 'inhalation injury', 'days after burns' |
| 0.8644 | 0.0307 | 'deep partial thickness burns', 'local_deep partial thickness burns', 'local_full thickness burns', 'inhalation injury' |
| 0.8279 | 0.0367 | 'deep partial thickness burns', 'local_full thickness burns', 'inhalation injury' |
| 0.7509 | 0.0510 | 'deep partial thickness burns', 'inhalation injury' |
| 0.5085 | 0.0619 | 'deep partial thickness burns' |

## 2. Supplementary Figures

**
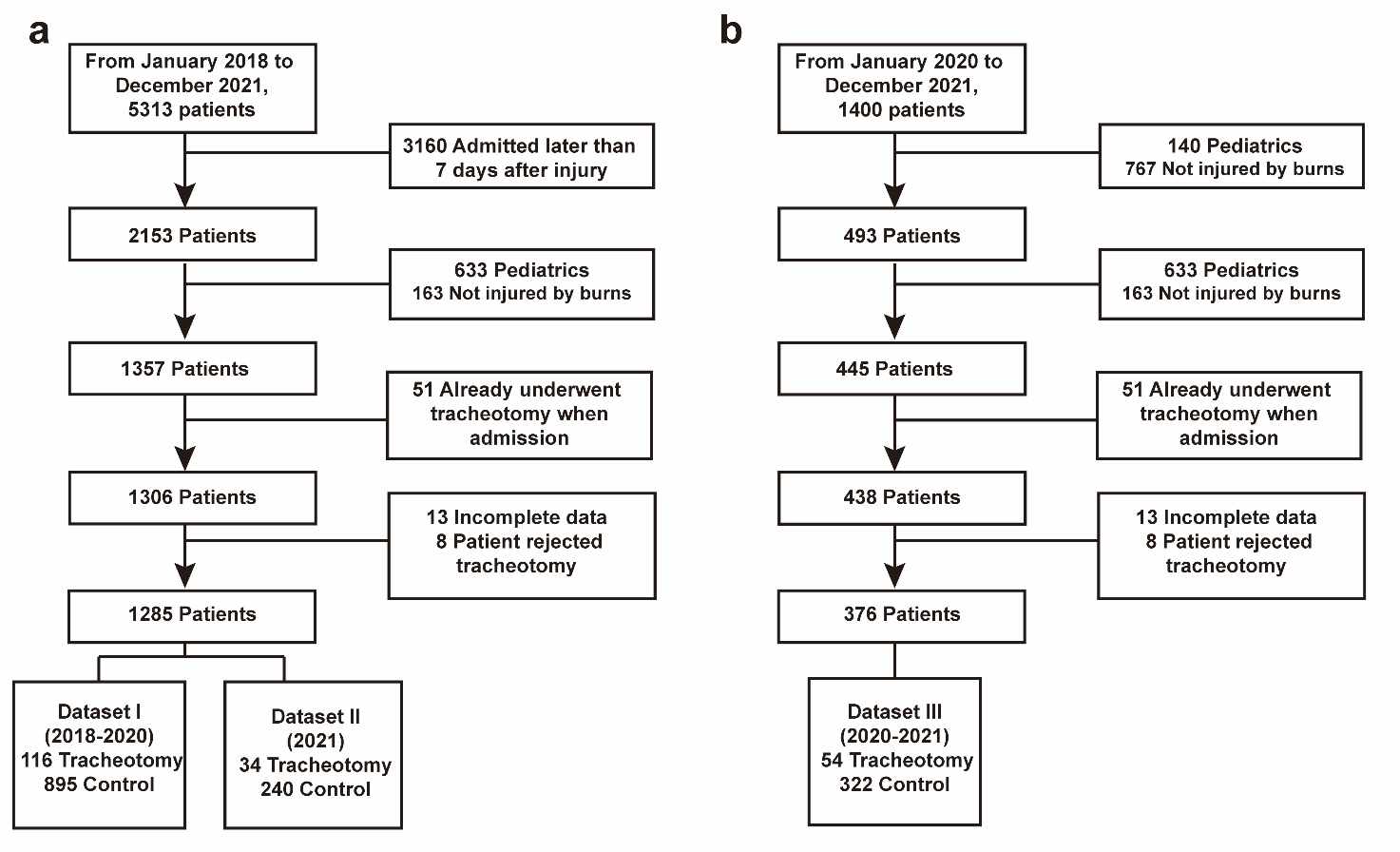
**

**Supplementary Figure 1. The flowchart of patient inclusion and exclusion in A) Hospital A and B) Hospital B**


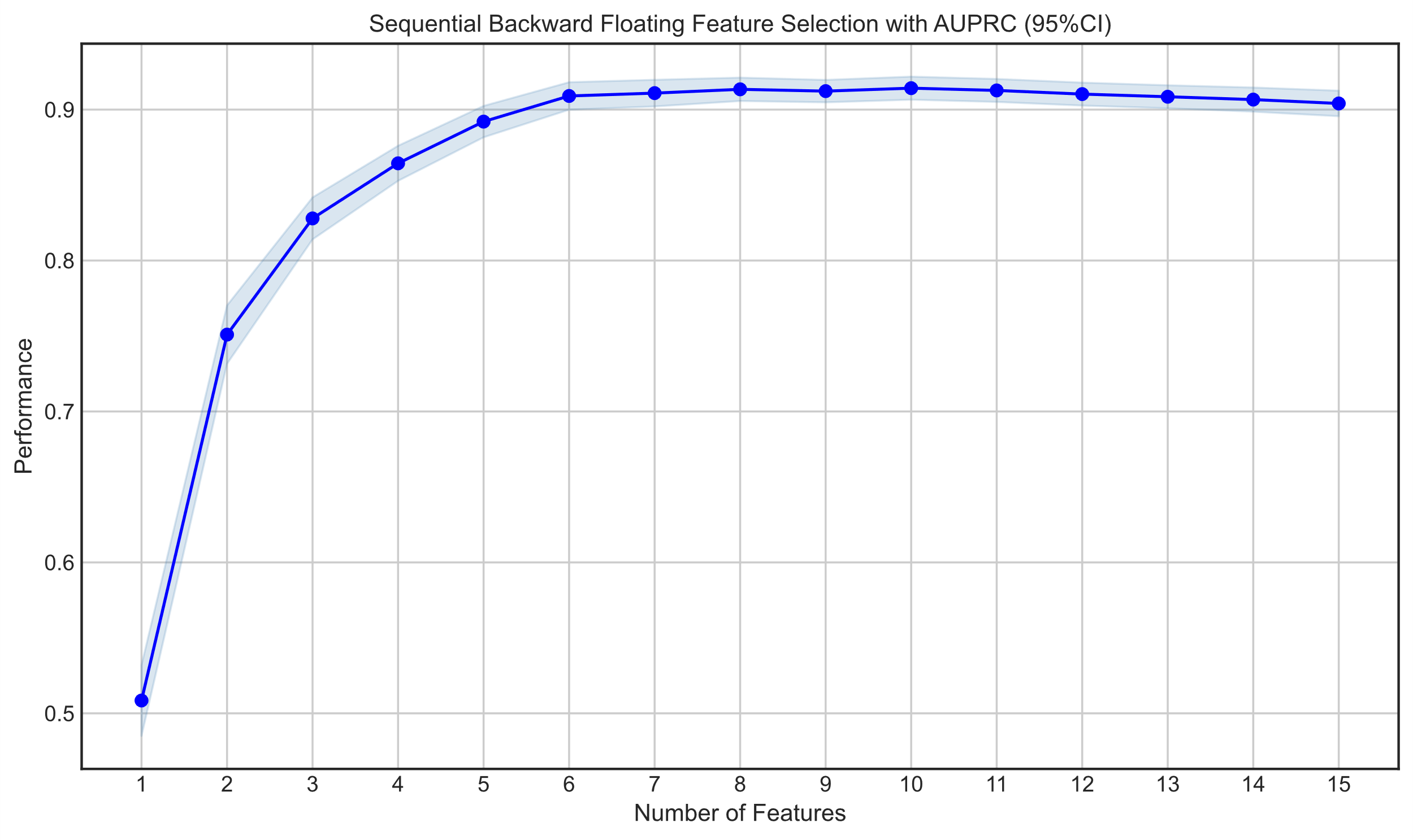


**Supplementary Figure 2. AUPRCs of XGB algorithms with different feature subsets by sequential backward floating selection (SBFS).**

**
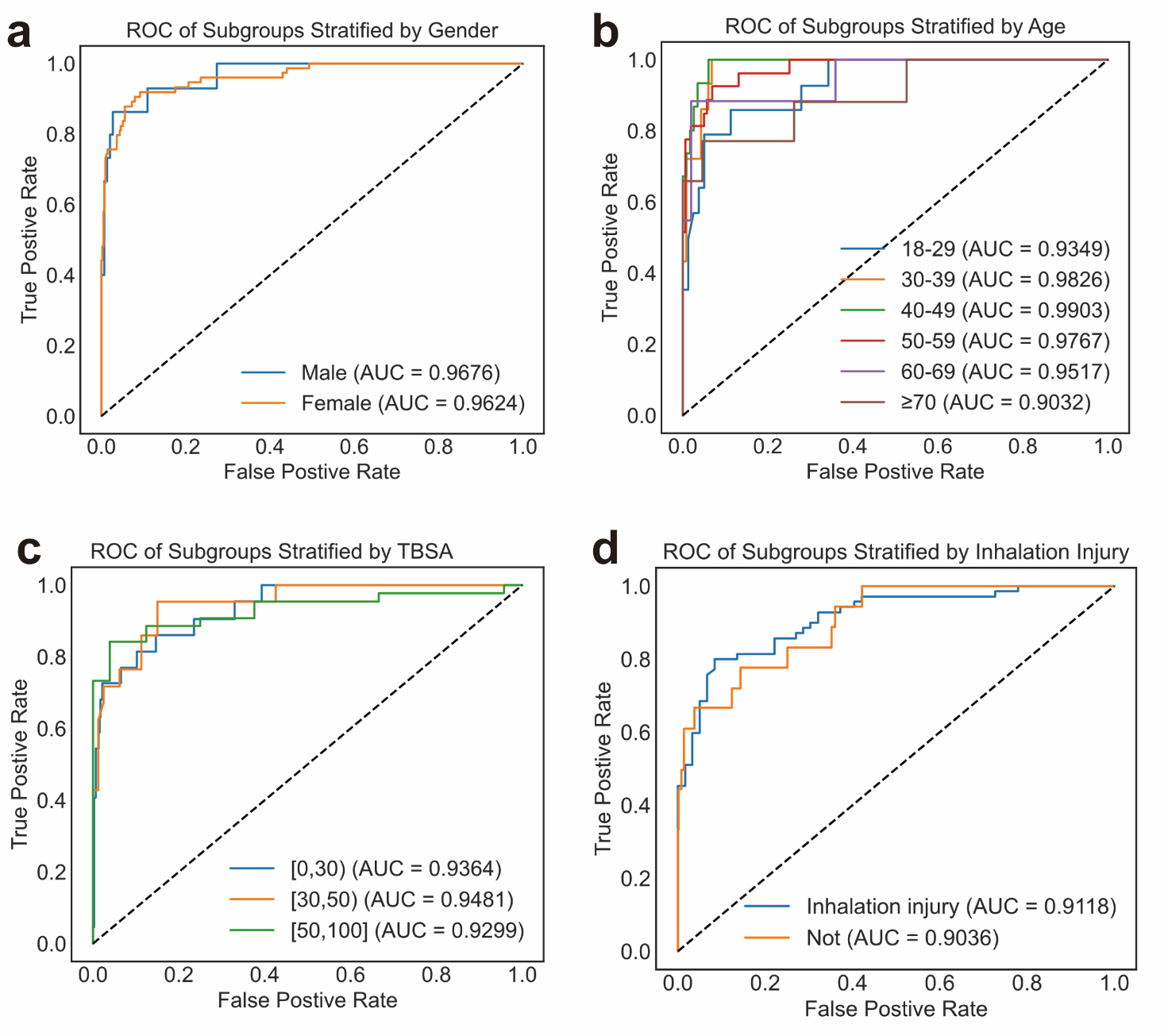
Supplementary Figure 3. Comparative performances of the final model in different subgroups stratified by sex, age, total burn area, and inhalation injury**.

**A.** ROC of the final model in subgroups stratified by sex.

**B.** ROC of the final model in subgroups stratified by age.

**C.** ROC of the final model in subgroups stratified by total burn area.

**D.** ROC of the final model in subgroups stratified by inhalation injury.

AUC=area under the curves; ROC= receiver operating characteristic curve; TBSA=total body surface area.

**
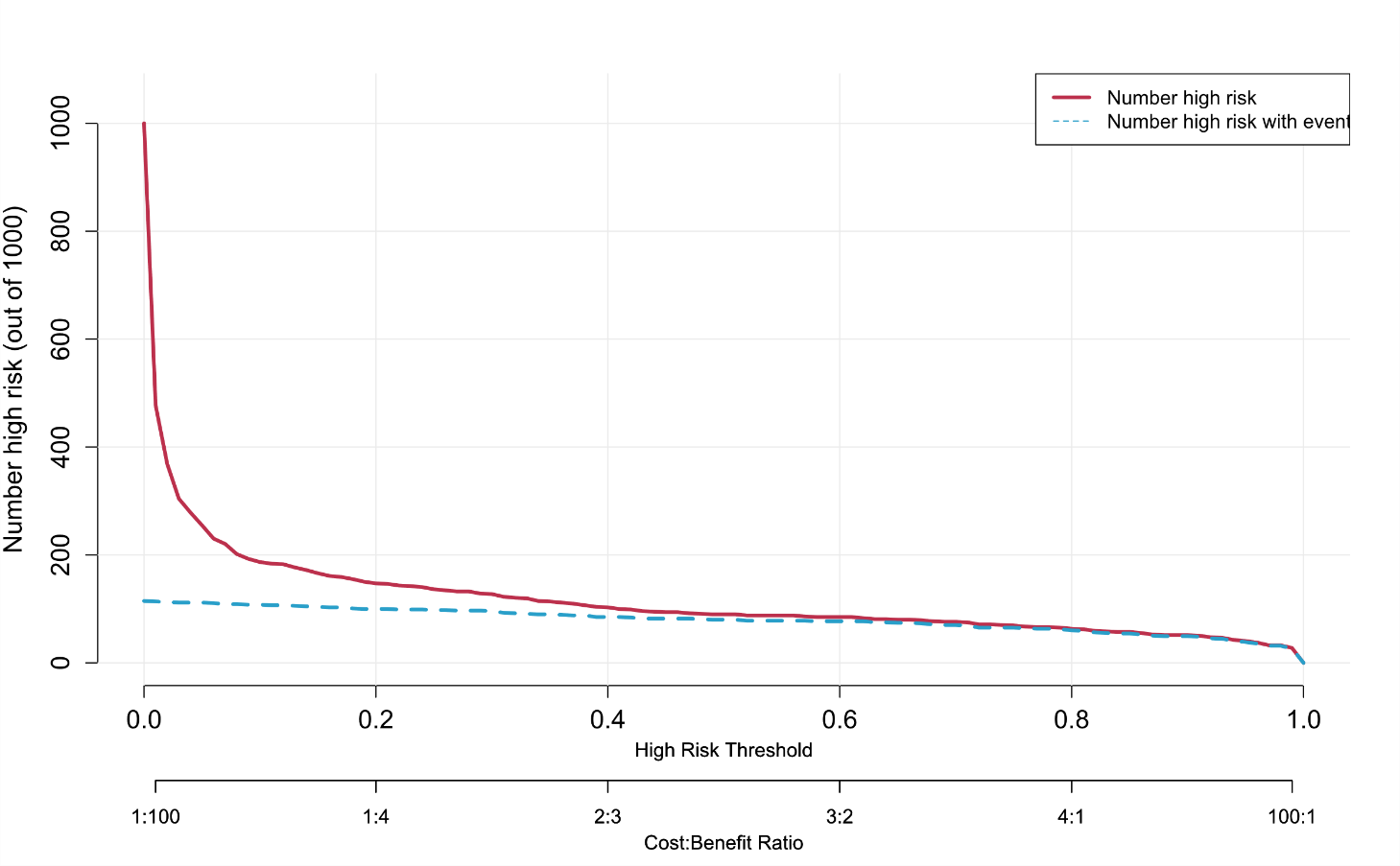
**

**Supplementary Figure 4. The clinical impact curve of the developed nomogram**


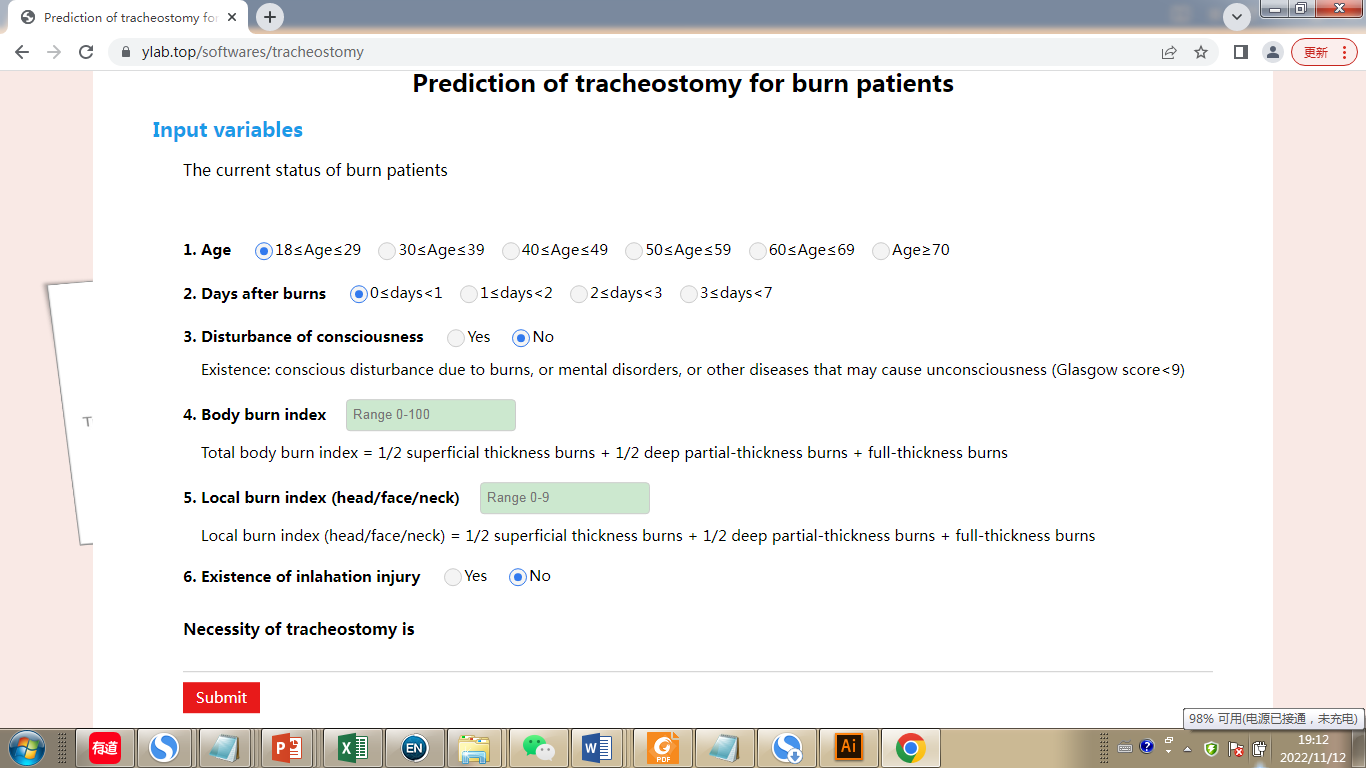


**Supplementary Figure 5. The developed online calculator**(https://lihaisheng-burns.github.io/tracheostomy-burn/)
